# Crystal structure of [tris­(pyridin-2-ylmeth­yl)amine-κ^4^ *N*]copper(II) bromide

**DOI:** 10.1107/S2056989016007568

**Published:** 2016-05-10

**Authors:** Emma C. Bridgman, Megan M. Doherty, Kaleigh A. Ellis, Elizabeth A. Homer, Taylor N. Lashbrook, Margaret E. Mraz, Gina C. Pernesky, Emma M. Vreeke, Kayode D. Oshin, Allen G. Oliver

**Affiliations:** a Department of Chemistry and Physics, Saint Mary’s College, Notre Dame, IN 46556, USA; b Department of Chemistry and Biochemistry, University of Notre Dame, Notre Dame, IN 46556, USA

**Keywords:** crystal structure, five-coordinate copper(II) complex, Atom Transfer Radical Addition (ATRA), ligand disorder

## Abstract

In the asymmetric unit of the title compound, [CuBr(C_18_H_18_N_4_)]Br, there are three crystallographically independent cations. One of the cations exhibits positional disorder of the pyridin-2-yl­methyl groups over two sets of sites with refined occupancies of 0.672 (8) and 0.328 (8). The outer-sphere bromine counter-ion is severely disordered over multiple sites. In each cation, the Cu^II^ ion is coordinated by the four N atoms of the tris­(pyridin-2-ylmeth­yl)amine ligand and one bromine and adopts a slightly distorted trigonal–bipyramidal geometry.

## Abstract 2

The complex [tris­(pyridin-2-ylmeth­yl)amine]­copper(II) bromide adopts a trigonal–bipyramidal coordination geometry about the Cu^II^ ion. The outer sphere bromine counter-ions are severely disordered.

## Chemical context   

Atom Transfer Radical Addition (ATRA) reactions involve the formation of carbon–carbon bonds through the addition of saturated poly-halogenated hydro­carbons to alkenes (Eckenhoff & Pintauer, 2010 ▸). First reported by Kharasch in the 1940s (Kharasch *et al.*, 1945 ▸), the reaction incorporates halogen-group functionalities within products which can be used as starting reagents in further functionalization reactions (Iqbal *et al.*, 1994 ▸). Subsequently, ATRA reactions have emerged as some of the most atom-economical methods for simultaneously forming C—C and C—*X* bonds, leading to the production of more attractive mol­ecules (Eckenhoff & Pintauer, 2010 ▸). Most ATRA reactions proceed in the presence of a free-radical precursor or transition metal complex (catalyst), as the halogen-atom transfer agent and have been efficiently catalyzed by complexes incorporating nickel, ruthenium, iron, and copper (Eckenhoff *et al.*, 2008 ▸). Studies suggest that the type of ligands used in ATRA reactions significantly influence the behavior of the catalyst generated due to different steric and electronic inter­actions with the metal atom (Matyjaszewski *et al.*, 2001 ▸). Copper complexes made with tetra­dentate nitro­gen-based ligands such as tris­[2-(di­methyl­amino)­eth­yl]amine (Me_6_TREN), 1,4,8,11-tetra­aza-1,4,8,11-tetra­methyl­cyclo­tetra­decane (Me_6_CYCLAM), and tris­(pyridin-2-yl­meth­yl)amine (TPMA) are currently some of the most active multi-dentate ligand structures used in atom-transfer radical reactions (Tang *et al.*, 2008 ▸). Given the significance and application of complexes made from these tetra­dentate ligands, we report on the synthesis and crystal structure of the title compound [CuBr(C_18_H_18_N_4_)]Br (I) which incorporates tris(pyridin-2-yl­meth­yl)amine.
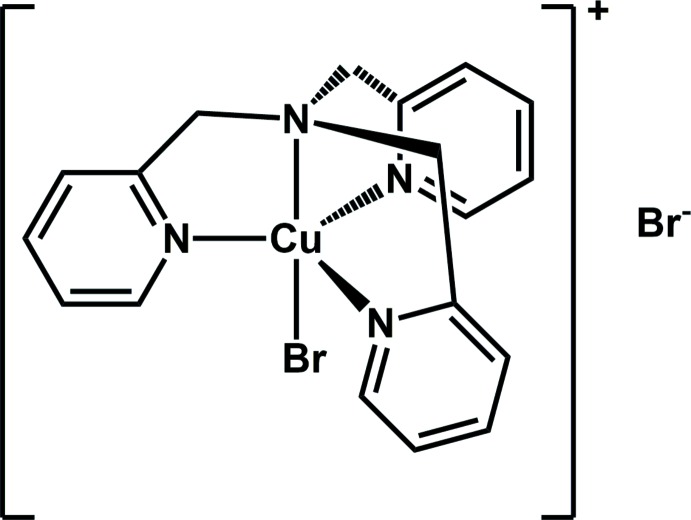


## Structural commentary   

**Figure 1:**
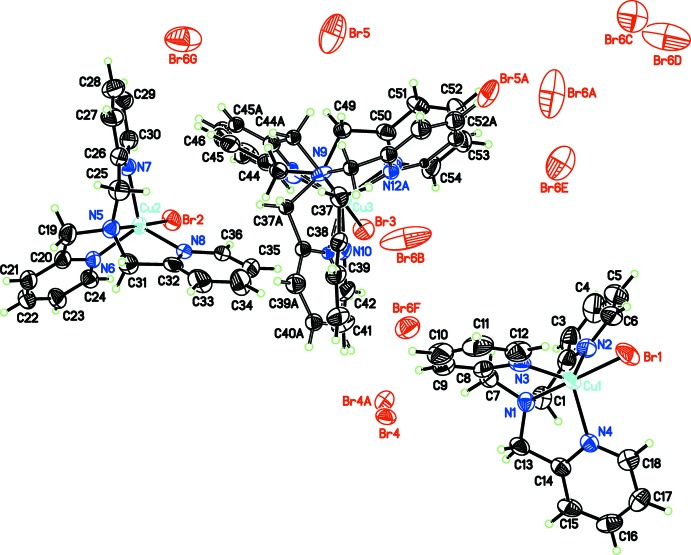
Labeling scheme for [tris­(pyridin-2-ylmeth­yl)amine]­copper(II) bromide. Atomic displacement ellipsoids depicted at 50% probability and H atoms as spheres of arbitrary radius. Some labels are omitted for clarity.

There are three crystallographically independent copper(II) atoms within the asymmetric unit reported herein (Fig. 1 ▸). Each of the atoms adopts a slightly distorted trigonal–bipyramidal geometry and is coordinated by the four nitro­gen atoms of the tris­(pyridin-2-yl­meth­yl)amine ligand and one bromine atom (Table 1 ▸). The amine nitro­gen and bromine atoms adopt the apical positions of the coordination environment and the pyridine nitro­gen atoms are located in the equatorial plane. Derived metrics (bond lengths and angles) from the copper atoms to their respective coordinating atoms are typical (*MOGUL* analysis; Bruno *et al.*, 2004 ▸). The τ-5 values for Cu1, Cu2 and Cu3 are 0.99, 0.99 and 0.89, respectively (Addison *et al.*, 1984 ▸); the latter deviates the most from ideal geometry due to the disorder present in that mol­ecule.

One of the three independent cations exhibits positional disorder of the pyridin-2-yl­methyl groups (see *Refinement* below for specific details). Despite this disorder, the connectivity is unequivocal. Unlike the polymorphic structure (Eckenhoff *et al.*, 2008 ▸) that has crystallographically imposed symmetry on the pyridin-2-yl­methyl arms, the pyridin-2-yl­methyl groups on the cations reported here have geometries independent of the others. Furthermore, the structure here is mixture of Δ and Λ conformations of the ligand, whereas Eckenhoff’s structure has chirally resolved upon crystallization.

## Supra­molecular features   

**Figure 2:**
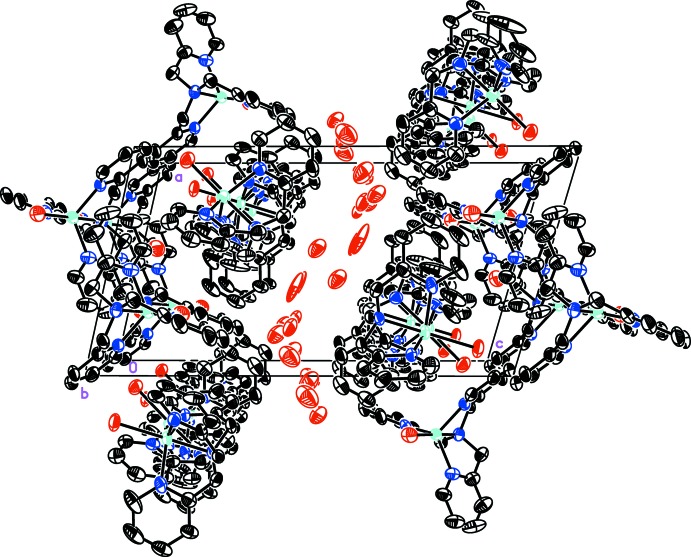
Packing diagram of [tris­(pyridin-2-ylmeth­yl)amine]­copper(II) bromide, viewed along the *b* axis, highlighting the channels in which disordered bromine ions reside. H atoms and the minor disorder components are omitted for clarity. Atomic displacement parameters depicted at 50% probability.

The prominent feature of the crystal packing within this structure is the excessive positional disorder of the outer-sphere bromine anions. These are observed in a channel within the lattice (Fig. 2 ▸) that presumably has unresolvable solvent of crystallization also present. Because there are no prominent charge surfaces, packing is solely due to van der Waals inter­actions.

## Database survey   

There are six reported copper(II) bromide structures deposited in the Cambridge Structure Database incorporating the tris­(pyridin-2-yl­meth­yl)amine ligand derivatives (Groom *et al.*, 2016 ▸; CSD Version 5.37 plus one update). Of those six structures, one is a dimer incorporating two bridging bromine ligands (Maiti *et al.*, 2007 ▸) and the remaining five are monomers. Out of the five monomer structures, three incorporate methyl or meth­oxy electron-withdrawing groups (Kaur *et al.*, 2015 ▸), while one incorporates hydroxyl electron-donating groups (He *et al.*, 2000 ▸). The final structure is a polymorph of that presented here: it incorporates an unsubstituted TPMA ligand framework but adopts a different space group (cubic, *P*2_1_3) and unit-cell parameters (*a* = 12.633 Å) due to lack of disorder in the ligand framework (Eckenhoff *et al.*, 2008 ▸). Of the six total reported structures, four adopt similar distorted five-coordinate geometries as observed in complex (I), while two adopt a distorted six-coordinate geometry about the metal atom.

## Synthesis and crystallization   

**Synthesis of tris­(pyridin-2-yl­meth­yl)amine (TPMA) ligand:** the TPMA ligand was synthesized according to modified literature procedures (Britovsek *et al.*, 2005 ▸). A 500 mL round-bottom flask was charged with 100 mL of di­chloro­methane solvent. While mixing, 2-(amino­meth­yl)pyridine (1.62 mL, 15.0 mmol) and sodium tri­acet­oxy­borohydride (9.63 g, 44.2 mmol) were added, generating a clear-colored solution. 2-Pyridine­carboxaldehyde (3.38 g, 31.54 mmol) was slowly added to the mixture, producing a yellow-colored solution. The reaction was allowed to mix for 24 h and inter­rupted with the addition of sodium hydrogen carbonate until a pH of 10 was achieved. Extractions were performed on the resulting solution with ethyl acetate and the organic layers collected. The organic layer was subsequently dried using magnesium sulfate (MgSO_4_) and solvent removed using a rotary evaporator to generate a yellow residue. This residue was dried under vacuum for three h to produce the desired ligand as a yellow solid (4.43 g, 97%). ^1^H NMR (CDCl_3_, 400 MHz): δ3.86 (*s*, 2H), δ7.51 (*d*, 1H), δ7.63 (*t*, 1H), δ 8.52 (*d*, 1H). ^13^C NMR (CDCl_3_, 400 MHz): δ 60.60, 122.35, 123.32, 136.59, 149.35, 159.81. FT–IR (solid) *v* (cm^−1^): 3048 (*s*), 3009 (*s*), 2920 (*s*), 2803 (*s*), 1585 (*s*), 1566 (*s*), 970 (*s*), 745 (*s*).
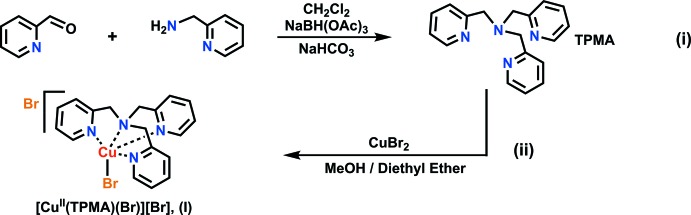


**Synthesis of tris(pyridin-2-yl­meth­yl)amine copper(II) bromide complex:** TPMA (0.500 g, 1.72 mmol) was dissolved in 15 mL methanol in a 100 mL round-bottom flask. Copper(II) bromide (0.384 g, 1.72 mmol) was added to the flask to give a greenish-blue-colored solution. The reaction was allowed to mix for one hour then 30 mL of diethyl ether was transferred into the flask, facilitating the precipitation of the desired complex as a green powder. The mixture was filtered and the precipitate washed with excess diethyl ether solvent. The precipitate was dried under vacuum for 30 minutes to yield a green-colored solid (1.44 g, 94%). TOF–ESI–MS: (*m*/*z*) [*M* – (Br)]^+^ calculated for C_18_H_18_N_4_CuBr = 432.00, found 432.03. FT–IR (solid): *v* (cm^−1^) = 3337 (*b*), 2018 (*s*), 1600 (*s*), 1473 (*s*), 1426 (*s*), 1257 (*s*), 1150 (*s*), 1015 (*s*), 949 (*s*), 837 (s). UV–Vis: λ_max_ (MeOH) = 700 nm. Green-colored single crystals suitable for X-ray analysis were obtained by slow diffusion of diethyl ether into a concentrated complex solution made in methanol.

## Refinement   

Crystal data, data collection and structure refinement details are summarized in Table 2 ▸. The two ordered cations, the major occupancy component of the disordered cation and all outer-sphere bromine atoms were modeled with anisotropic atomic displacement parameters. The minor occupancy component of the disordered cation was modeled with isotropic atomic displacement parameters. Hydrogen atoms were included in geometrically calculated positions with C—H = 0.99 (methyl­ene) and 0.95 Å (aromatic) and *U*_iso_(H) = 1.2*U*_eq_(C).

The disorder of the pyridin-2-yl­methyl groups was observed as residual electron density oriented in approximately a mirror to the major occupancy components. The occupancies of the two components were refined summed to unity, yielding an approximately 0.67:0.33 ratio. The pyridine rings for both components were constrained to an ideal hexa­gon, with C—C = 1.39 Å.

All of the outer-sphere, non-coordinating bromine counter-ions were found to be disordered over multiple sites. Initially, occupancies were refined freely to identify possible site pairings. One bromine (Br4) was found to be nearly fully located at one site. In subsequent refinement cycles, residual density adjacent to the site was revealed and ultimately modeled as a bromine disordered over two sites with occupancies 0.80:0.20. Two bromine sites whose occupancies refined independently to nearly 50% were both set to 50% occupancy and assumed to be disorder of the same bromine atom (Br5/5*A*). Final residual electron density ranging from 8 to 13 e Å^−3^ was observed. Because an additional bromine was required for charge balance and there were no other counter-ions used during synthesis, it was assumed that the final bromine was disordered over multiple sites, presumably in concert with solvent from crystallization. Ultimately, seven locations were refined as partial-occupancy bromine atoms with a total occupancy summed to unity, yielding a 0.13:0.17:0.17:0.20:0.11:0.12:0.10 ratio of sites. The solvent contribution could not be reliably modeled.

## Supplementary Material

Crystal structure: contains datablock(s) I. DOI: 10.1107/S2056989016007568/lh5812sup1.cif

Structure factors: contains datablock(s) I. DOI: 10.1107/S2056989016007568/lh5812Isup2.hkl

CCDC reference: 1478413

Additional supporting information:  crystallographic information; 3D view; checkCIF report

## Figures and Tables

**Table 1:** Selected geometric parameters (Å, °)

Cu1—N3	2.037 (7)	Cu2—N6	2.071 (6)
Cu1—N1	2.054 (6)	Cu2—Br2	2.3664 (12)
Cu1—N4	2.060 (6)	Cu3—N11	2.004 (10)
Cu1—N2	2.060 (7)	Cu3—N10	2.045 (7)
Cu1—Br1	2.3781 (12)	Cu3—N9	2.046 (6)
Cu2—N5	2.035 (6)	Cu3—N12	2.115 (6)
Cu2—N7	2.060 (6)	Cu3—Br3	2.3715 (11)
Cu2—N8	2.061 (7)		
			
N3—Cu1—N1	81.5 (3)	N8—Cu2—N6	118.1 (2)
N3—Cu1—N4	119.7 (3)	N5—Cu2—Br2	177.8 (2)
N1—Cu1—N4	80.4 (3)	N7—Cu2—Br2	97.18 (19)
N3—Cu1—N2	120.1 (3)	N8—Cu2—Br2	98.5 (2)
N1—Cu1—N2	81.5 (3)	N6—Cu2—Br2	100.86 (19)
N4—Cu1—N2	113.3 (3)	N11—Cu3—N10	126.2 (4)
N3—Cu1—Br1	98.9 (2)	N11—Cu3—N9	82.6 (3)
N1—Cu1—Br1	179.4 (2)	N10—Cu3—N9	81.3 (3)
N4—Cu1—Br1	99.82 (18)	N11—Cu3—N12	118.1 (4)
N2—Cu1—Br1	97.8 (2)	N10—Cu3—N12	110.5 (4)
N5—Cu2—N7	81.3 (3)	N9—Cu3—N12	83.3 (3)
N5—Cu2—N8	81.0 (3)	N11—Cu3—Br3	97.3 (3)
N7—Cu2—N8	118.7 (3)	N10—Cu3—Br3	98.4 (2)
N5—Cu2—N6	81.2 (3)	N9—Cu3—Br3	179.7 (2)
N7—Cu2—N6	116.3 (2)	N12—Cu3—Br3	97.0 (2)

**Table 2:** Experimental details

Crystal data	
Chemical formula	[CuBr(C_18_H_18_N_4_)]Br
*M*_r_	513.72
Crystal system, space group	Triclinic, *P* 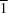
Temperature (K)	120
*a*, *b*, *c* (Å)	11.5415 (7), 15.2747 (9), 19.9663 (12)
α, β, γ (°)	88.425 (2), 75.894 (2), 69.650 (2)
*V* (Å^3^)	3194.4 (3)
*Z*	6
Radiation type	Mo *K*α
μ (mm^−1^)	4.79
Crystal size (mm)	0.30 × 0.30 × 0.26

Data collection	
Diffractometer	Bruker APEXII
Absorption correction	Multi-scan (*SADABS*; Bruker, 2015 ▸)
*T*_min_, *T*_max_	0.688, 0.862
No. of measured, independent and observed [*I* > 2σ(*I*)] reflections	25682, 13032, 10550
*R*_int_	0.020
(sin θ/λ)_max_ (Å^−1^)	0.626

Refinement	
*R*[*F* ^2^ > 2σ(*F* ^2^)], *wR*(*F* ^2^), *S*	0.086, 0.254, 1.04
No. of reflections	13032
No. of parameters	769
No. of restraints	1
H-atom treatment	H-atom parameters constrained
Δρ_max_, Δρ_min_ (e Å^−3^)	3.78, −1.50

Computer programs: *APEX3* and *SAINT* (Bruker, 2015 ▸), *SHELXT2014/2* (Sheldrick, 2015*a* ▸), *SHELXL2014/7* (Sheldrick, 2015*b* ▸), *XP* (Bruker, 2015 ▸) and *publCIF* (Westrip, 2010 ▸).

## supplementary crystallographic information

### Crystal data

**Table:**

[CuBr(C_18_H_18_N_4_)]Br	*Z* = 6
*M**_r_* = 513.72	*F*(000) = 1518
Triclinic, *P*1	*D*_x_ = 1.602 Mg m^−^^3^
*a* = 11.5415 (7) Å	Mo *K*α radiation, λ = 0.71073 Å
*b* = 15.2747 (9) Å	Cell parameters from 9798 reflections
*c* = 19.9663 (12) Å	θ = 2.4–26.3°
α = 88.425 (2)°	µ = 4.79 mm^−^^1^
β = 75.894 (2)°	*T* = 120 K
γ = 69.650 (2)°	Block, green
*V* = 3194.4 (3) Å^3^	0.30 × 0.30 × 0.26 mm

### Data collection

**Table:**

Bruker APEXII diffractometer	13032 independent reflections
Radiation source: fine-focus sealed tube	10550 reflections with *I* > 2σ(*I*)
Detector resolution: 8.33 pixels mm^-1^	*R*_int_ = 0.020
combination of ω and φ–scans	θ_max_ = 26.4°, θ_min_ = 1.4°
Absorption correction: multi-scan (*SADABS*; Bruker, 2015)	*h* = −14→14
*T*_min_ = 0.688, *T*_max_ = 0.862	*k* = −19→19
25682 measured reflections	*l* = −24→24

### Refinement

**Table:**

Refinement on *F*^2^	Primary atom site location: real-space vector search
Least-squares matrix: full	Secondary atom site location: difference Fourier map
*R*[*F*^2^ > 2σ(*F*^2^)] = 0.086	Hydrogen site location: inferred from neighbouring sites
*wR*(*F*^2^) = 0.254	H-atom parameters constrained
*S* = 1.04	*w* = 1/[σ^2^(*F*_o_^2^) + (0.1449*P*)^2^ + 25.6015*P*] where *P* = (*F*_o_^2^ + 2*F*_c_^2^)/3
13032 reflections	(Δ/σ)_max_ = 0.027
769 parameters	Δρ_max_ = 3.78 e Å^−^^3^
1 restraint	Δρ_min_ = −1.50 e Å^−^^3^

### Special details

**Table:**

Geometry. All esds (except the esd in the dihedral angle between two l.s. planes) are estimated using the full covariance matrix. The cell esds are taken into account individually in the estimation of esds in distances, angles and torsion angles; correlations between esds in cell parameters are only used when they are defined by crystal symmetry. An approximate (isotropic) treatment of cell esds is used for estimating esds involving l.s. planes.
Refinement. The outer sphere bromine anion atoms were all found to be disordered over multiple sites. Br4/4A was found to occupy two sites close to each other and was refined with occupancies summed to unity yielding an approximate 0.83:0.17 ratio. Br5/5A was modeled as two half occupancy bromine atoms from an initial, independent, refinement of the occupancies for these sites. Br6 is disordered over multiple sites. Occupancies of the sites were refined summed to unity yielding an approximately 0.14:0.17:0.17:0.20:0.11:0.12:0.09 ratio of site occupancies. Attempts to model this disorder as undifferentiated solvent did not meet with success. Furthermore, because the electron density associated with this is located within the enveloped developed by SQUEEZE, this routine could not be employed. The result is that there is some additional residual electron density that cannot be reliably accounted for. Presumably there is solvent of crystallization present at the sites when they are not occupied by anions. This was not modeled.

### Fractional atomic coordinates and isotropic or equivalent isotropic displacement parameters (Å^2^)

**Table:**

	*x*	*y*	*z*	*U*_iso_*/*U*_eq_	Occ. (<1)
Cu1	0.29355 (9)	0.21210 (6)	0.12631 (5)	0.0349 (2)	
Br1	0.28112 (9)	0.08631 (6)	0.19713 (5)	0.0542 (3)	
N1	0.3062 (6)	0.3198 (4)	0.0645 (3)	0.0357 (14)	
N2	0.4733 (6)	0.1456 (5)	0.0635 (4)	0.0431 (16)	
N3	0.2663 (7)	0.3099 (5)	0.2015 (4)	0.0418 (16)	
N4	0.1495 (6)	0.2241 (4)	0.0789 (3)	0.0337 (13)	
C1	0.3979 (9)	0.2781 (6)	−0.0032 (4)	0.046 (2)	
H1A	0.4339	0.3246	−0.0260	0.055*	
H1B	0.3527	0.2608	−0.0340	0.055*	
C2	0.5051 (8)	0.1917 (6)	0.0084 (5)	0.046 (2)	
C3	0.6262 (10)	0.1613 (8)	−0.0350 (6)	0.063 (3)	
H3	0.6477	0.1957	−0.0734	0.076*	
C4	0.7151 (10)	0.0802 (9)	−0.0217 (8)	0.080 (4)	
H4	0.7995	0.0590	−0.0506	0.096*	
C5	0.6831 (10)	0.0305 (8)	0.0323 (7)	0.069 (3)	
H5	0.7430	−0.0270	0.0405	0.082*	
C6	0.5597 (8)	0.0659 (7)	0.0757 (5)	0.051 (2)	
H6	0.5368	0.0326	0.1146	0.061*	
C7	0.3496 (9)	0.3801 (6)	0.1005 (4)	0.0426 (18)	
H7A	0.3277	0.4429	0.0819	0.051*	
H7B	0.4434	0.3530	0.0934	0.051*	
C8	0.2855 (8)	0.3877 (6)	0.1763 (4)	0.0413 (18)	
C9	0.2535 (10)	0.4680 (7)	0.2179 (5)	0.057 (2)	
H9	0.2657	0.5227	0.1988	0.068*	
C10	0.2035 (11)	0.4664 (8)	0.2878 (5)	0.063 (3)	
H10	0.1798	0.5206	0.3176	0.075*	
C11	0.1881 (11)	0.3863 (9)	0.3143 (5)	0.065 (3)	
H11	0.1563	0.3841	0.3626	0.078*	
C12	0.2189 (10)	0.3083 (8)	0.2701 (5)	0.055 (2)	
H12	0.2065	0.2533	0.2884	0.066*	
C13	0.1770 (8)	0.3701 (6)	0.0540 (4)	0.0396 (17)	
H13A	0.1834	0.4080	0.0131	0.048*	
H13B	0.1233	0.4128	0.0949	0.048*	
C14	0.1174 (8)	0.3004 (5)	0.0431 (4)	0.0365 (16)	
C15	0.0359 (9)	0.3132 (7)	0.0003 (4)	0.047 (2)	
H15	0.0122	0.3689	−0.0235	0.056*	
C16	−0.0100 (8)	0.2432 (7)	−0.0068 (4)	0.050 (2)	
H16	−0.0625	0.2485	−0.0379	0.060*	
C17	0.0196 (9)	0.1657 (7)	0.0305 (4)	0.0453 (19)	
H17	−0.0135	0.1176	0.0267	0.054*	
C18	0.0975 (8)	0.1589 (6)	0.0734 (4)	0.0407 (18)	
H18	0.1159	0.1061	0.1005	0.049*	
Cu2	0.78574 (9)	1.03414 (6)	0.22801 (5)	0.0341 (2)	
Br2	0.94552 (9)	0.97310 (6)	0.12441 (4)	0.0507 (3)	
N5	0.6517 (6)	1.0824 (5)	0.3189 (3)	0.0366 (14)	
N6	0.7077 (6)	1.1724 (4)	0.2059 (3)	0.0346 (13)	
N7	0.9054 (6)	1.0078 (4)	0.2935 (3)	0.0345 (13)	
N8	0.6826 (7)	0.9480 (4)	0.2270 (4)	0.0428 (16)	
C19	0.6282 (9)	1.1832 (6)	0.3303 (5)	0.046 (2)	
H19A	0.6920	1.1906	0.3528	0.055*	
H19B	0.5424	1.2139	0.3615	0.055*	
C20	0.6366 (7)	1.2294 (5)	0.2629 (4)	0.0374 (17)	
C21	0.5785 (8)	1.3247 (6)	0.2589 (5)	0.047 (2)	
H21	0.5261	1.3640	0.2990	0.056*	
C22	0.5998 (9)	1.3609 (6)	0.1940 (5)	0.051 (2)	
H22	0.5631	1.4263	0.1899	0.061*	
C23	0.6724 (9)	1.3040 (6)	0.1362 (5)	0.048 (2)	
H23	0.6865	1.3288	0.0920	0.058*	
C24	0.7246 (8)	1.2095 (6)	0.1439 (5)	0.0415 (18)	
H24	0.7743	1.1691	0.1040	0.050*	
C25	0.7049 (8)	1.0265 (7)	0.3737 (4)	0.0457 (19)	
H25A	0.6898	0.9664	0.3753	0.055*	
H25B	0.6613	1.0611	0.4194	0.055*	
C26	0.8459 (8)	1.0079 (6)	0.3590 (4)	0.0407 (18)	
C27	0.9092 (10)	0.9863 (7)	0.4114 (5)	0.052 (2)	
H27	0.8644	0.9841	0.4578	0.062*	
C28	1.0400 (11)	0.9678 (8)	0.3942 (6)	0.061 (3)	
H28	1.0858	0.9550	0.4291	0.074*	
C29	1.1022 (10)	0.9683 (7)	0.3262 (6)	0.057 (2)	
H29	1.1919	0.9542	0.3135	0.068*	
C30	1.0333 (9)	0.9894 (6)	0.2762 (5)	0.0443 (19)	
H30	1.0761	0.9910	0.2293	0.053*	
C31	0.5351 (8)	1.0677 (6)	0.3124 (5)	0.048 (2)	
H31A	0.4885	1.1177	0.2860	0.057*	
H31B	0.4784	1.0701	0.3589	0.057*	
C32	0.5716 (8)	0.9739 (6)	0.2755 (6)	0.055 (3)	
C33	0.4913 (10)	0.9229 (8)	0.2883 (10)	0.093 (5)	
H33	0.4125	0.9444	0.3224	0.112*	
C34	0.5301 (13)	0.8390 (9)	0.2492 (12)	0.128 (8)	
H34	0.4785	0.8011	0.2567	0.154*	
C35	0.6430 (12)	0.8119 (7)	0.2002 (9)	0.085 (5)	
H35	0.6709	0.7546	0.1733	0.102*	
C36	0.7173 (11)	0.8676 (6)	0.1895 (6)	0.058 (3)	
H36	0.7951	0.8483	0.1545	0.069*	
Cu3	0.78179 (9)	0.55333 (7)	0.25199 (4)	0.0332 (2)	
Br3	0.84691 (9)	0.53090 (6)	0.12962 (4)	0.0474 (2)	
N9	0.7248 (6)	0.5733 (5)	0.3575 (3)	0.0336 (13)	
C37	0.6067 (11)	0.5541 (8)	0.3805 (6)	0.036 (3)	0.672 (8)
H37A	0.5608	0.5841	0.4274	0.043*	0.672 (8)
H37B	0.6266	0.4858	0.3830	0.043*	0.672 (8)
C38	0.5254 (6)	0.5897 (6)	0.3329 (3)	0.040 (3)	0.672 (8)
C39	0.3929 (7)	0.6198 (7)	0.3517 (4)	0.048 (3)	0.672 (8)
H39	0.3490	0.6207	0.3987	0.058*	0.672 (8)
C40	0.3247 (6)	0.6484 (7)	0.3016 (6)	0.059 (4)	0.672 (8)
H40	0.2341	0.6690	0.3144	0.071*	0.672 (8)
C41	0.3889 (10)	0.6470 (9)	0.2328 (5)	0.073 (9)	0.672 (8)
H41	0.3423	0.6666	0.1986	0.087*	0.672 (8)
C42	0.5214 (10)	0.6170 (8)	0.2140 (3)	0.064 (11)	0.672 (8)
H42	0.5653	0.6160	0.1670	0.077*	0.672 (8)
N10	0.5897 (6)	0.5883 (6)	0.2641 (4)	0.036 (2)	0.672 (8)
C43	0.7222 (10)	0.6622 (8)	0.3746 (6)	0.033 (2)	0.672 (8)
H43A	0.7308	0.6644	0.4226	0.040*	0.672 (8)
H43B	0.6388	0.7092	0.3727	0.040*	0.672 (8)
C44	0.8253 (9)	0.6864 (6)	0.3276 (4)	0.036 (3)	0.672 (8)
C45	0.8754 (10)	0.7504 (6)	0.3455 (5)	0.047 (3)	0.672 (8)
H45	0.8435	0.7812	0.3904	0.056*	0.672 (8)
C46	0.9721 (10)	0.7694 (7)	0.2977 (7)	0.049 (5)	0.672 (8)
H46	1.0064	0.8131	0.3099	0.059*	0.672 (8)
C47	1.0188 (10)	0.7243 (10)	0.2320 (6)	0.043 (5)	0.672 (8)
H47	1.0849	0.7373	0.1993	0.052*	0.672 (8)
C48	0.9687 (12)	0.6603 (10)	0.2141 (4)	0.043 (5)	0.672 (8)
H48	1.0005	0.6295	0.1691	0.052*	0.672 (8)
N11	0.8719 (11)	0.6413 (7)	0.2618 (5)	0.038 (4)	0.672 (8)
C49	0.8310 (12)	0.4950 (8)	0.3817 (6)	0.035 (2)	0.672 (8)
H49A	0.9063	0.5142	0.3762	0.042*	0.672 (8)
H49B	0.8000	0.4871	0.4315	0.042*	0.672 (8)
C50	0.8702 (8)	0.4041 (4)	0.3422 (3)	0.036 (3)	0.672 (8)
C51	0.9133 (9)	0.3163 (5)	0.3678 (3)	0.041 (3)	0.672 (8)
H51	0.9169	0.3109	0.4148	0.049*	0.672 (8)
C52	0.9512 (9)	0.2362 (4)	0.3246 (5)	0.046 (3)	0.672 (8)
H52	0.9807	0.1762	0.3421	0.055*	0.672 (8)
C53	0.9460 (11)	0.2441 (5)	0.2558 (4)	0.045 (4)	0.672 (8)
H53	0.9719	0.1894	0.2263	0.053*	0.672 (8)
C54	0.9028 (12)	0.3319 (7)	0.2302 (3)	0.051 (5)	0.672 (8)
H54	0.8992	0.3373	0.1831	0.061*	0.672 (8)
N12	0.8649 (10)	0.4120 (5)	0.2734 (4)	0.031 (2)	0.672 (8)
C37A	0.5973 (18)	0.6729 (13)	0.3737 (10)	0.023 (4)*	0.328 (8)
H37C	0.5488	0.6781	0.4226	0.028*	0.328 (8)
H37D	0.6252	0.7275	0.3653	0.028*	0.328 (8)
C38A	0.5180 (11)	0.6709 (10)	0.3282 (6)	0.025 (4)*	0.328 (8)
C39A	0.3857 (11)	0.7107 (10)	0.3429 (6)	0.040 (6)*	0.328 (8)
H39A	0.3389	0.7423	0.3867	0.048*	0.328 (8)
C40A	0.3221 (9)	0.7041 (12)	0.2935 (7)	0.030 (5)*	0.328 (8)
H40A	0.2317	0.7313	0.3036	0.036*	0.328 (8)
C41A	0.3907 (12)	0.6578 (13)	0.2294 (6)	0.026 (7)*	0.328 (8)
H41A	0.3472	0.6534	0.1957	0.031*	0.328 (8)
C42A	0.5229 (12)	0.6180 (10)	0.2147 (5)	0.012 (8)*	0.328 (8)
H42A	0.5698	0.5864	0.1709	0.015*	0.328 (8)
N10A	0.5866 (9)	0.6246 (8)	0.2641 (6)	0.017 (4)*	0.328 (8)
C43A	0.8177 (18)	0.6049 (14)	0.3841 (10)	0.023 (4)*	0.328 (8)
H43C	0.7719	0.6422	0.4285	0.028*	0.328 (8)
H43D	0.8860	0.5489	0.3934	0.028*	0.328 (8)
C44A	0.8751 (18)	0.6595 (12)	0.3373 (8)	0.032 (6)*	0.328 (8)
C45A	0.9284 (19)	0.7224 (13)	0.3536 (7)	0.026 (5)*	0.328 (8)
H45A	0.9212	0.7376	0.4006	0.031*	0.328 (8)
C46A	0.992 (2)	0.7630 (15)	0.3010 (11)	0.041 (9)*	0.328 (8)
H46A	1.0286	0.8060	0.3122	0.049*	0.328 (8)
C47A	1.003 (2)	0.7407 (19)	0.2323 (9)	0.031 (8)*	0.328 (8)
H47A	1.0463	0.7685	0.1964	0.037*	0.328 (8)
C48A	0.949 (3)	0.6778 (18)	0.2160 (7)	0.019 (6)*	0.328 (8)
H48A	0.9565	0.6626	0.1690	0.023*	0.328 (8)
N11A	0.886 (2)	0.6372 (13)	0.2685 (10)	0.016 (6)*	0.328 (8)
C49A	0.6874 (19)	0.5069 (14)	0.3899 (10)	0.023 (4)*	0.328 (8)
H49C	0.5988	0.5162	0.3885	0.028*	0.328 (8)
H49D	0.6915	0.5074	0.4389	0.028*	0.328 (8)
C50A	0.7774 (15)	0.4172 (9)	0.3521 (7)	0.028 (4)*	0.328 (8)
C51A	0.8117 (16)	0.3309 (10)	0.3808 (6)	0.042 (6)*	0.328 (8)
H51A	0.7801	0.3263	0.4289	0.051*	0.328 (8)
C52A	0.8923 (17)	0.2511 (8)	0.3392 (8)	0.040 (7)*	0.328 (8)
H52A	0.9157	0.1920	0.3588	0.048*	0.328 (8)
C53A	0.9385 (18)	0.2577 (10)	0.2688 (8)	0.027 (6)*	0.328 (8)
H53A	0.9935	0.2031	0.2403	0.032*	0.328 (8)
C54A	0.9041 (17)	0.3441 (13)	0.2401 (6)	0.009 (4)*	0.328 (8)
H54A	0.9357	0.3486	0.1920	0.011*	0.328 (8)
N12A	0.8236 (15)	0.4238 (10)	0.2818 (7)	0.027 (6)*	0.328 (8)
Br4	0.2753 (9)	0.6342 (2)	0.06977 (13)	0.0424 (9)	0.80 (4)
Br4A	0.320 (5)	0.626 (2)	0.0748 (10)	0.046 (6)	0.20 (4)
Br5	0.9243 (4)	0.4418 (2)	0.53149 (17)	0.1024 (11)	0.5
Br5A	0.66590 (18)	0.18484 (14)	0.53556 (10)	0.0538 (5)	0.5
Br6A	0.6049 (11)	0.0709 (6)	0.5606 (3)	0.083 (4)	0.136 (3)
Br6B	0.3821 (5)	0.4517 (7)	0.4322 (2)	0.085 (3)	0.171 (3)
Br6C	0.8053 (6)	−0.1392 (5)	0.5725 (4)	0.059 (2)	0.167 (3)
Br6D	0.7729 (6)	−0.1886 (7)	0.5463 (3)	0.090 (3)	0.198 (3)
Br6E	0.4331 (9)	0.1069 (7)	0.5323 (4)	0.053 (3)	0.107 (3)
Br6F	0.5711 (7)	0.5148 (6)	0.0703 (4)	0.051 (2)	0.125 (3)
Br6G	1.0726 (13)	0.7636 (11)	0.4667 (8)	0.078 (4)	0.096 (3)

### Atomic displacement parameters (Å^2^)

**Table:**

	*U*^11^	*U*^22^	*U*^33^	*U*^12^	*U*^13^	*U*^23^
Cu1	0.0329 (5)	0.0306 (5)	0.0386 (5)	−0.0070 (4)	−0.0112 (4)	0.0110 (4)
Br1	0.0519 (5)	0.0450 (5)	0.0618 (6)	−0.0129 (4)	−0.0160 (4)	0.0285 (4)
N1	0.044 (4)	0.033 (3)	0.032 (3)	−0.016 (3)	−0.009 (3)	0.005 (3)
N2	0.034 (3)	0.041 (4)	0.052 (4)	−0.010 (3)	−0.011 (3)	0.000 (3)
N3	0.043 (4)	0.047 (4)	0.038 (4)	−0.011 (3)	−0.023 (3)	0.010 (3)
N4	0.036 (3)	0.033 (3)	0.029 (3)	−0.009 (3)	−0.008 (2)	0.002 (2)
C1	0.049 (5)	0.049 (5)	0.038 (4)	−0.022 (4)	0.000 (4)	0.001 (4)
C2	0.043 (5)	0.044 (5)	0.053 (5)	−0.021 (4)	−0.006 (4)	−0.004 (4)
C3	0.060 (6)	0.059 (6)	0.068 (7)	−0.031 (5)	0.007 (5)	−0.011 (5)
C4	0.039 (5)	0.065 (7)	0.117 (11)	−0.016 (5)	0.015 (6)	−0.020 (7)
C5	0.043 (5)	0.056 (6)	0.094 (9)	−0.001 (5)	−0.015 (5)	−0.006 (6)
C6	0.038 (4)	0.047 (5)	0.063 (6)	−0.007 (4)	−0.016 (4)	−0.001 (4)
C7	0.051 (5)	0.037 (4)	0.046 (5)	−0.018 (4)	−0.018 (4)	0.011 (3)
C8	0.046 (4)	0.043 (4)	0.045 (5)	−0.017 (4)	−0.027 (4)	0.008 (3)
C9	0.067 (6)	0.054 (6)	0.055 (6)	−0.014 (5)	−0.034 (5)	−0.003 (4)
C10	0.074 (7)	0.069 (7)	0.047 (5)	−0.014 (5)	−0.033 (5)	−0.008 (5)
C11	0.064 (6)	0.094 (9)	0.039 (5)	−0.018 (6)	−0.026 (5)	−0.004 (5)
C12	0.065 (6)	0.069 (6)	0.039 (5)	−0.023 (5)	−0.027 (4)	0.011 (4)
C13	0.047 (4)	0.035 (4)	0.033 (4)	−0.005 (3)	−0.017 (3)	0.005 (3)
C14	0.040 (4)	0.036 (4)	0.028 (4)	−0.006 (3)	−0.010 (3)	0.002 (3)
C15	0.050 (5)	0.054 (5)	0.031 (4)	−0.005 (4)	−0.020 (4)	0.009 (4)
C16	0.041 (5)	0.073 (6)	0.035 (4)	−0.015 (4)	−0.014 (4)	−0.007 (4)
C17	0.048 (5)	0.056 (5)	0.034 (4)	−0.025 (4)	−0.004 (3)	−0.008 (4)
C18	0.041 (4)	0.047 (5)	0.031 (4)	−0.015 (4)	−0.003 (3)	−0.002 (3)
Cu2	0.0355 (5)	0.0312 (5)	0.0343 (5)	−0.0097 (4)	−0.0087 (4)	−0.0037 (4)
Br2	0.0523 (5)	0.0471 (5)	0.0380 (5)	−0.0038 (4)	−0.0037 (4)	−0.0084 (4)
N5	0.033 (3)	0.039 (3)	0.038 (3)	−0.014 (3)	−0.007 (3)	−0.002 (3)
N6	0.030 (3)	0.030 (3)	0.042 (4)	−0.008 (2)	−0.009 (3)	−0.006 (3)
N7	0.035 (3)	0.034 (3)	0.038 (3)	−0.016 (3)	−0.011 (3)	0.003 (3)
N8	0.046 (4)	0.026 (3)	0.064 (5)	−0.007 (3)	−0.034 (4)	0.003 (3)
C19	0.046 (5)	0.043 (5)	0.045 (5)	−0.014 (4)	−0.007 (4)	−0.011 (4)
C20	0.032 (4)	0.035 (4)	0.047 (4)	−0.014 (3)	−0.008 (3)	−0.002 (3)
C21	0.038 (4)	0.040 (4)	0.058 (5)	−0.008 (4)	−0.011 (4)	−0.011 (4)
C22	0.049 (5)	0.034 (4)	0.070 (6)	−0.010 (4)	−0.020 (4)	0.000 (4)
C23	0.050 (5)	0.034 (4)	0.059 (5)	−0.013 (4)	−0.013 (4)	0.009 (4)
C24	0.037 (4)	0.039 (4)	0.045 (5)	−0.011 (3)	−0.007 (3)	0.001 (3)
C25	0.042 (4)	0.051 (5)	0.038 (4)	−0.013 (4)	−0.005 (3)	0.000 (4)
C26	0.047 (5)	0.036 (4)	0.042 (4)	−0.014 (3)	−0.017 (4)	0.001 (3)
C27	0.063 (6)	0.054 (5)	0.038 (5)	−0.014 (4)	−0.023 (4)	0.011 (4)
C28	0.076 (7)	0.062 (6)	0.065 (7)	−0.031 (5)	−0.042 (6)	0.015 (5)
C29	0.054 (6)	0.057 (6)	0.073 (7)	−0.026 (5)	−0.032 (5)	0.014 (5)
C30	0.048 (5)	0.039 (4)	0.056 (5)	−0.024 (4)	−0.020 (4)	0.011 (4)
C31	0.030 (4)	0.043 (5)	0.066 (6)	−0.013 (3)	−0.007 (4)	0.001 (4)
C32	0.034 (4)	0.035 (4)	0.110 (8)	−0.011 (4)	−0.044 (5)	0.011 (5)
C33	0.031 (5)	0.054 (6)	0.200 (17)	−0.011 (5)	−0.045 (7)	−0.001 (8)
C34	0.057 (8)	0.049 (7)	0.30 (3)	−0.019 (6)	−0.089 (12)	−0.016 (10)
C35	0.069 (8)	0.040 (5)	0.159 (14)	−0.002 (5)	−0.073 (9)	−0.022 (7)
C36	0.071 (6)	0.029 (4)	0.084 (7)	−0.006 (4)	−0.058 (6)	0.000 (4)
Cu3	0.0383 (5)	0.0402 (5)	0.0208 (4)	−0.0115 (4)	−0.0105 (3)	0.0043 (3)
Br3	0.0607 (5)	0.0542 (5)	0.0224 (4)	−0.0158 (4)	−0.0081 (3)	0.0019 (3)
N9	0.034 (3)	0.051 (4)	0.023 (3)	−0.020 (3)	−0.014 (2)	0.004 (3)
C37	0.042 (6)	0.035 (6)	0.026 (5)	−0.012 (5)	−0.003 (4)	−0.002 (4)
C38	0.044 (6)	0.034 (6)	0.042 (7)	−0.010 (5)	−0.018 (5)	−0.008 (5)
C39	0.037 (6)	0.046 (7)	0.060 (8)	−0.006 (5)	−0.019 (6)	−0.013 (6)
C40	0.043 (8)	0.054 (9)	0.085 (12)	−0.012 (6)	−0.033 (8)	−0.007 (8)
C41	0.078 (14)	0.065 (12)	0.100 (18)	−0.026 (9)	−0.067 (13)	0.001 (10)
C42	0.10 (2)	0.043 (10)	0.078 (16)	−0.032 (9)	−0.067 (14)	0.009 (7)
N10	0.053 (6)	0.028 (6)	0.033 (5)	−0.014 (4)	−0.021 (4)	−0.005 (4)
C43	0.034 (6)	0.033 (6)	0.029 (5)	−0.005 (4)	−0.011 (4)	−0.002 (4)
C44	0.049 (7)	0.031 (6)	0.030 (6)	−0.009 (5)	−0.018 (5)	0.004 (4)
C45	0.035 (7)	0.042 (7)	0.057 (8)	0.002 (6)	−0.020 (6)	−0.007 (6)
C46	0.031 (7)	0.030 (7)	0.082 (13)	−0.005 (5)	−0.013 (7)	−0.002 (6)
C47	0.024 (6)	0.032 (7)	0.065 (10)	0.001 (6)	−0.014 (6)	0.018 (6)
C48	0.032 (8)	0.030 (8)	0.054 (9)	0.006 (6)	−0.014 (6)	0.016 (5)
N11	0.040 (7)	0.038 (7)	0.028 (6)	−0.003 (5)	−0.008 (5)	0.000 (4)
C49	0.047 (6)	0.031 (5)	0.027 (5)	−0.010 (5)	−0.018 (5)	0.002 (4)
C50	0.038 (6)	0.036 (6)	0.028 (5)	−0.002 (5)	−0.012 (4)	0.000 (4)
C51	0.041 (7)	0.043 (7)	0.034 (6)	−0.008 (5)	−0.010 (5)	0.013 (5)
C52	0.049 (8)	0.036 (6)	0.041 (7)	−0.011 (6)	0.001 (6)	0.005 (5)
C53	0.041 (7)	0.034 (7)	0.054 (8)	−0.010 (5)	−0.008 (6)	−0.007 (6)
C54	0.051 (9)	0.049 (9)	0.052 (9)	−0.016 (7)	−0.011 (7)	−0.018 (7)
N12	0.032 (6)	0.035 (6)	0.025 (5)	−0.008 (5)	−0.010 (4)	0.005 (4)
Br4	0.043 (2)	0.0419 (7)	0.0400 (7)	−0.0046 (8)	−0.0224 (8)	0.0031 (5)
Br4A	0.055 (12)	0.051 (6)	0.040 (4)	−0.028 (7)	−0.011 (5)	0.002 (4)
Br5	0.119 (2)	0.106 (2)	0.0821 (19)	−0.060 (2)	0.0087 (17)	−0.0372 (17)
Br5A	0.0488 (10)	0.0572 (11)	0.0579 (11)	−0.0253 (8)	−0.0048 (8)	−0.0256 (9)
Br6A	0.164 (9)	0.078 (5)	0.015 (3)	−0.090 (6)	0.034 (4)	−0.004 (3)
Br6B	0.037 (3)	0.206 (9)	0.010 (2)	−0.043 (4)	−0.0021 (18)	0.001 (3)
Br6C	0.053 (3)	0.075 (4)	0.048 (4)	−0.036 (3)	0.006 (3)	0.021 (3)
Br6D	0.054 (3)	0.139 (7)	0.023 (2)	0.023 (4)	0.004 (2)	−0.005 (3)
Br6E	0.056 (5)	0.070 (6)	0.042 (4)	−0.044 (5)	0.006 (4)	0.001 (4)
Br6F	0.048 (4)	0.060 (5)	0.048 (4)	−0.017 (3)	−0.018 (3)	−0.007 (3)
Br6G	0.070 (8)	0.102 (10)	0.088 (9)	−0.050 (7)	−0.042 (7)	0.051 (8)

### Geometric parameters (Å, º)

**Table:**

Cu1—N3	2.037 (7)	C36—H36	0.9500
Cu1—N1	2.054 (6)	Cu3—N12A	1.977 (12)
Cu1—N4	2.060 (6)	Cu3—N11	2.004 (10)
Cu1—N2	2.060 (7)	Cu3—N10	2.045 (7)
Cu1—Br1	2.3781 (12)	Cu3—N9	2.046 (6)
N1—C7	1.465 (11)	Cu3—N10A	2.088 (9)
N1—C13	1.485 (10)	Cu3—N11A	2.114 (17)
N1—C1	1.495 (10)	Cu3—N12	2.115 (6)
N2—C2	1.335 (12)	Cu3—Br3	2.3715 (11)
N2—C6	1.339 (11)	N9—C49A	1.33 (2)
N3—C8	1.346 (11)	N9—C43	1.398 (13)
N3—C12	1.347 (12)	N9—C37	1.456 (13)
N4—C14	1.337 (10)	N9—C43A	1.52 (2)
N4—C18	1.348 (11)	N9—C49	1.551 (12)
C1—C2	1.521 (13)	N9—C37A	1.68 (2)
C1—H1A	0.9900	C37—C38	1.459 (13)
C1—H1B	0.9900	C37—H37A	0.9900
C2—C3	1.379 (13)	C37—H37B	0.9900
C3—C4	1.374 (18)	C38—C39	1.3900
C3—H3	0.9500	C38—N10	1.3900
C4—C5	1.353 (18)	C39—C40	1.3900
C4—H4	0.9500	C39—H39	0.9500
C5—C6	1.401 (14)	C40—C41	1.3900
C5—H5	0.9500	C40—H40	0.9500
C6—H6	0.9500	C41—C42	1.3900
C7—C8	1.502 (12)	C41—H41	0.9500
C7—H7A	0.9900	C42—N10	1.3900
C7—H7B	0.9900	C42—H42	0.9500
C8—C9	1.388 (13)	C43—C44	1.475 (14)
C9—C10	1.377 (15)	C43—H43A	0.9900
C9—H9	0.9500	C43—H43B	0.9900
C10—C11	1.370 (17)	C44—C45	1.3900
C10—H10	0.9500	C44—N11	1.3900
C11—C12	1.391 (15)	C45—C46	1.3900
C11—H11	0.9500	C45—H45	0.9500
C12—H12	0.9500	C46—C47	1.3900
C13—C14	1.499 (12)	C46—H46	0.9500
C13—H13A	0.9900	C47—C48	1.3900
C13—H13B	0.9900	C47—H47	0.9500
C14—C15	1.382 (11)	C48—N11	1.3900
C15—C16	1.373 (14)	C48—H48	0.9500
C15—H15	0.9500	C49—C50	1.485 (12)
C16—C17	1.367 (14)	C49—H49A	0.9900
C16—H16	0.9500	C49—H49B	0.9900
C17—C18	1.363 (12)	C50—C51	1.3900
C17—H17	0.9500	C50—N12	1.3900
C18—H18	0.9500	C51—C52	1.3900
Cu2—N5	2.035 (6)	C51—H51	0.9500
Cu2—N7	2.060 (6)	C52—C53	1.3900
Cu2—N8	2.061 (7)	C52—H52	0.9500
Cu2—N6	2.071 (6)	C53—C54	1.3900
Cu2—Br2	2.3664 (12)	C53—H53	0.9500
N5—C31	1.475 (11)	C54—N12	1.3900
N5—C19	1.482 (11)	C54—H54	0.9500
N5—C25	1.488 (11)	C37A—C38A	1.45 (2)
N6—C24	1.346 (11)	C37A—H37C	0.9900
N6—C20	1.352 (10)	C37A—H37D	0.9900
N7—C26	1.320 (11)	C38A—C39A	1.3900
N7—C30	1.360 (11)	C38A—N10A	1.3900
N8—C32	1.341 (13)	C39A—C40A	1.3900
N8—C36	1.339 (11)	C39A—H39A	0.9500
C19—C20	1.500 (12)	C40A—C41A	1.3900
C19—H19A	0.9900	C40A—H40A	0.9500
C19—H19B	0.9900	C41A—C42A	1.3900
C20—C21	1.384 (12)	C41A—H41A	0.9500
C21—C22	1.395 (14)	C42A—N10A	1.3900
C21—H21	0.9500	C42A—H42A	0.9500
C22—C23	1.367 (14)	C43A—C44A	1.43 (2)
C22—H22	0.9500	C43A—H43C	0.9900
C23—C24	1.377 (12)	C43A—H43D	0.9900
C23—H23	0.9500	C44A—C45A	1.3900
C24—H24	0.9500	C44A—N11A	1.3900
C25—C26	1.505 (12)	C45A—C46A	1.3900
C25—H25A	0.9900	C45A—H45A	0.9500
C25—H25B	0.9900	C46A—C47A	1.3900
C26—C27	1.387 (12)	C46A—H46A	0.9500
C27—C28	1.390 (15)	C47A—C48A	1.3900
C27—H27	0.9500	C47A—H47A	0.9500
C28—C29	1.374 (16)	C48A—N11A	1.3900
C28—H28	0.9500	C48A—H48A	0.9500
C29—C30	1.387 (13)	C49A—C50A	1.49 (2)
C29—H29	0.9500	C49A—H49C	0.9900
C30—H30	0.9500	C49A—H49D	0.9900
C31—C32	1.503 (13)	C50A—C51A	1.3900
C31—H31A	0.9900	C50A—N12A	1.3900
C31—H31B	0.9900	C51A—C52A	1.3900
C32—C33	1.380 (14)	C51A—H51A	0.9500
C33—C34	1.39 (2)	C52A—C53A	1.3900
C33—H33	0.9500	C52A—H52A	0.9500
C34—C35	1.36 (2)	C53A—C54A	1.3900
C34—H34	0.9500	C53A—H53A	0.9500
C35—C36	1.384 (17)	C54A—N12A	1.3900
C35—H35	0.9500	C54A—H54A	0.9500
			
N3—Cu1—N1	81.5 (3)	N11—Cu3—N10	126.2 (4)
N3—Cu1—N4	119.7 (3)	N12A—Cu3—N9	77.5 (5)
N1—Cu1—N4	80.4 (3)	N11—Cu3—N9	82.6 (3)
N3—Cu1—N2	120.1 (3)	N10—Cu3—N9	81.3 (3)
N1—Cu1—N2	81.5 (3)	N12A—Cu3—N10A	113.6 (6)
N4—Cu1—N2	113.3 (3)	N9—Cu3—N10A	80.5 (4)
N3—Cu1—Br1	98.9 (2)	N12A—Cu3—N11A	121.0 (8)
N1—Cu1—Br1	179.4 (2)	N9—Cu3—N11A	79.7 (6)
N4—Cu1—Br1	99.82 (18)	N10A—Cu3—N11A	115.0 (7)
N2—Cu1—Br1	97.8 (2)	N11—Cu3—N12	118.1 (4)
C7—N1—C13	111.9 (6)	N10—Cu3—N12	110.5 (4)
C7—N1—C1	112.2 (7)	N9—Cu3—N12	83.3 (3)
C13—N1—C1	110.0 (6)	N12A—Cu3—Br3	102.8 (5)
C7—N1—Cu1	107.6 (5)	N11—Cu3—Br3	97.3 (3)
C13—N1—Cu1	107.1 (5)	N10—Cu3—Br3	98.4 (2)
C1—N1—Cu1	107.9 (5)	N9—Cu3—Br3	179.7 (2)
C2—N2—C6	119.3 (8)	N10A—Cu3—Br3	99.2 (3)
C2—N2—Cu1	114.5 (6)	N11A—Cu3—Br3	100.3 (5)
C6—N2—Cu1	126.0 (7)	N12—Cu3—Br3	97.0 (2)
C8—N3—C12	119.1 (8)	C43—N9—C37	117.6 (8)
C8—N3—Cu1	113.2 (6)	C49A—N9—C43A	118.1 (12)
C12—N3—Cu1	127.2 (7)	C43—N9—C49	111.6 (7)
C14—N4—C18	118.0 (7)	C37—N9—C49	108.0 (8)
C14—N4—Cu1	114.7 (5)	C49A—N9—C37A	107.2 (12)
C18—N4—Cu1	126.8 (5)	C43A—N9—C37A	99.7 (10)
N1—C1—C2	109.9 (7)	C49A—N9—Cu3	114.9 (9)
N1—C1—H1A	109.7	C43—N9—Cu3	107.3 (6)
C2—C1—H1A	109.7	C37—N9—Cu3	107.8 (5)
N1—C1—H1B	109.7	C43A—N9—Cu3	109.9 (8)
C2—C1—H1B	109.7	C49—N9—Cu3	103.4 (5)
H1A—C1—H1B	108.2	C37A—N9—Cu3	105.0 (7)
N2—C2—C3	121.7 (9)	N9—C37—C38	110.6 (8)
N2—C2—C1	115.2 (7)	N9—C37—H37A	109.5
C3—C2—C1	123.1 (9)	C38—C37—H37A	109.5
C4—C3—C2	118.8 (11)	N9—C37—H37B	109.5
C4—C3—H3	120.6	C38—C37—H37B	109.5
C2—C3—H3	120.6	H37A—C37—H37B	108.1
C5—C4—C3	120.3 (10)	C39—C38—N10	120.0
C5—C4—H4	119.9	C39—C38—C37	124.6 (7)
C3—C4—H4	119.9	N10—C38—C37	115.3 (7)
C4—C5—C6	118.5 (10)	C40—C39—C38	120.0
C4—C5—H5	120.7	C40—C39—H39	120.0
C6—C5—H5	120.7	C38—C39—H39	120.0
N2—C6—C5	121.3 (10)	C39—C40—C41	120.0
N2—C6—H6	119.3	C39—C40—H40	120.0
C5—C6—H6	119.3	C41—C40—H40	120.0
N1—C7—C8	108.6 (7)	C42—C41—C40	120.0
N1—C7—H7A	110.0	C42—C41—H41	120.0
C8—C7—H7A	110.0	C40—C41—H41	120.0
N1—C7—H7B	110.0	N10—C42—C41	120.0
C8—C7—H7B	110.0	N10—C42—H42	120.0
H7A—C7—H7B	108.3	C41—C42—H42	120.0
N3—C8—C9	122.3 (9)	C42—N10—C38	120.0
N3—C8—C7	115.4 (7)	C42—N10—Cu3	127.7 (4)
C9—C8—C7	122.2 (8)	C38—N10—Cu3	112.1 (4)
C10—C9—C8	118.3 (10)	N9—C43—C44	112.2 (8)
C10—C9—H9	120.9	N9—C43—H43A	109.2
C8—C9—H9	120.9	C44—C43—H43A	109.2
C11—C10—C9	119.7 (10)	N9—C43—H43B	109.2
C11—C10—H10	120.2	C44—C43—H43B	109.2
C9—C10—H10	120.2	H43A—C43—H43B	107.9
C10—C11—C12	119.8 (10)	C45—C44—N11	120.0
C10—C11—H11	120.1	C45—C44—C43	123.8 (7)
C12—C11—H11	120.1	N11—C44—C43	116.2 (7)
N3—C12—C11	120.8 (10)	C46—C45—C44	120.0
N3—C12—H12	119.6	C46—C45—H45	120.0
C11—C12—H12	119.6	C44—C45—H45	120.0
N1—C13—C14	109.4 (6)	C45—C46—C47	120.0
N1—C13—H13A	109.8	C45—C46—H46	120.0
C14—C13—H13A	109.8	C47—C46—H46	120.0
N1—C13—H13B	109.8	C46—C47—C48	120.0
C14—C13—H13B	109.8	C46—C47—H47	120.0
H13A—C13—H13B	108.2	C48—C47—H47	120.0
N4—C14—C15	122.4 (8)	C47—C48—N11	120.0
N4—C14—C13	114.1 (7)	C47—C48—H48	120.0
C15—C14—C13	123.5 (7)	N11—C48—H48	120.0
C16—C15—C14	118.1 (8)	C48—N11—C44	120.0
C16—C15—H15	121.0	C48—N11—Cu3	129.4 (6)
C14—C15—H15	121.0	C44—N11—Cu3	110.6 (6)
C17—C16—C15	120.2 (8)	C50—C49—N9	112.4 (7)
C17—C16—H16	119.9	C50—C49—H49A	109.1
C15—C16—H16	119.9	N9—C49—H49A	109.1
C18—C17—C16	118.5 (8)	C50—C49—H49B	109.1
C18—C17—H17	120.7	N9—C49—H49B	109.1
C16—C17—H17	120.7	H49A—C49—H49B	107.9
N4—C18—C17	122.7 (8)	C51—C50—N12	120.0
N4—C18—H18	118.7	C51—C50—C49	125.7 (6)
C17—C18—H18	118.7	N12—C50—C49	114.3 (6)
N5—Cu2—N7	81.3 (3)	C52—C51—C50	120.0
N5—Cu2—N8	81.0 (3)	C52—C51—H51	120.0
N7—Cu2—N8	118.7 (3)	C50—C51—H51	120.0
N5—Cu2—N6	81.2 (3)	C51—C52—C53	120.0
N7—Cu2—N6	116.3 (2)	C51—C52—H52	120.0
N8—Cu2—N6	118.1 (2)	C53—C52—H52	120.0
N5—Cu2—Br2	177.8 (2)	C54—C53—C52	120.0
N7—Cu2—Br2	97.18 (19)	C54—C53—H53	120.0
N8—Cu2—Br2	98.5 (2)	C52—C53—H53	120.0
N6—Cu2—Br2	100.86 (19)	N12—C54—C53	120.0
C31—N5—C19	111.0 (7)	N12—C54—H54	120.0
C31—N5—C25	111.6 (7)	C53—C54—H54	120.0
C19—N5—C25	111.9 (7)	C54—N12—C50	120.0
C31—N5—Cu2	107.3 (5)	C54—N12—Cu3	128.6 (4)
C19—N5—Cu2	108.3 (5)	C50—N12—Cu3	111.2 (4)
C25—N5—Cu2	106.6 (5)	C38A—C37A—N9	108.1 (13)
C24—N6—C20	119.2 (7)	C38A—C37A—H37C	110.1
C24—N6—Cu2	127.7 (5)	N9—C37A—H37C	110.1
C20—N6—Cu2	113.0 (5)	C38A—C37A—H37D	110.1
C26—N7—C30	119.4 (7)	N9—C37A—H37D	110.1
C26—N7—Cu2	113.1 (5)	H37C—C37A—H37D	108.4
C30—N7—Cu2	127.5 (6)	C39A—C38A—N10A	120.0
C32—N8—C36	117.7 (8)	C39A—C38A—C37A	126.4 (11)
C32—N8—Cu2	113.4 (6)	N10A—C38A—C37A	113.6 (11)
C36—N8—Cu2	128.6 (7)	C38A—C39A—C40A	120.0
N5—C19—C20	110.5 (7)	C38A—C39A—H39A	120.0
N5—C19—H19A	109.5	C40A—C39A—H39A	120.0
C20—C19—H19A	109.5	C41A—C40A—C39A	120.0
N5—C19—H19B	109.5	C41A—C40A—H40A	120.0
C20—C19—H19B	109.5	C39A—C40A—H40A	120.0
H19A—C19—H19B	108.1	C40A—C41A—C42A	120.0
N6—C20—C21	121.7 (8)	C40A—C41A—H41A	120.0
N6—C20—C19	115.9 (7)	C42A—C41A—H41A	120.0
C21—C20—C19	122.4 (8)	N10A—C42A—C41A	120.0
C20—C21—C22	117.5 (8)	N10A—C42A—H42A	120.0
C20—C21—H21	121.3	C41A—C42A—H42A	120.0
C22—C21—H21	121.3	C42A—N10A—C38A	120.0
C23—C22—C21	121.2 (8)	C42A—N10A—Cu3	122.9 (7)
C23—C22—H22	119.4	C38A—N10A—Cu3	116.7 (7)
C21—C22—H22	119.4	C44A—C43A—N9	113.8 (15)
C22—C23—C24	117.9 (9)	C44A—C43A—H43C	108.8
C22—C23—H23	121.0	N9—C43A—H43C	108.8
C24—C23—H23	121.0	C44A—C43A—H43D	108.8
N6—C24—C23	122.4 (8)	N9—C43A—H43D	108.8
N6—C24—H24	118.8	H43C—C43A—H43D	107.7
C23—C24—H24	118.8	C45A—C44A—N11A	120.0
N5—C25—C26	110.0 (7)	C45A—C44A—C43A	127.1 (14)
N5—C25—H25A	109.7	N11A—C44A—C43A	112.6 (14)
C26—C25—H25A	109.7	C44A—C45A—C46A	120.0
N5—C25—H25B	109.7	C44A—C45A—H45A	120.0
C26—C25—H25B	109.7	C46A—C45A—H45A	120.0
H25A—C25—H25B	108.2	C47A—C46A—C45A	120.0
N7—C26—C27	122.8 (8)	C47A—C46A—H46A	120.0
N7—C26—C25	116.1 (7)	C45A—C46A—H46A	120.0
C27—C26—C25	121.0 (8)	C46A—C47A—C48A	120.0
C26—C27—C28	118.2 (9)	C46A—C47A—H47A	120.0
C26—C27—H27	120.9	C48A—C47A—H47A	120.0
C28—C27—H27	120.9	N11A—C48A—C47A	120.0
C29—C28—C27	119.2 (9)	N11A—C48A—H48A	120.0
C29—C28—H28	120.4	C47A—C48A—H48A	120.0
C27—C28—H28	120.4	C48A—N11A—C44A	120.0
C28—C29—C30	119.6 (10)	C48A—N11A—Cu3	123.9 (11)
C28—C29—H29	120.2	C44A—N11A—Cu3	115.7 (11)
C30—C29—H29	120.2	N9—C49A—C50A	105.6 (14)
N7—C30—C29	120.7 (9)	N9—C49A—H49C	110.6
N7—C30—H30	119.6	C50A—C49A—H49C	110.6
C29—C30—H30	119.6	N9—C49A—H49D	110.6
N5—C31—C32	109.1 (7)	C50A—C49A—H49D	110.6
N5—C31—H31A	109.9	H49C—C49A—H49D	108.7
C32—C31—H31A	109.9	C51A—C50A—N12A	120.0
N5—C31—H31B	109.9	C51A—C50A—C49A	125.5 (12)
C32—C31—H31B	109.9	N12A—C50A—C49A	114.5 (12)
H31A—C31—H31B	108.3	C52A—C51A—C50A	120.0
N8—C32—C33	123.8 (10)	C52A—C51A—H51A	120.0
N8—C32—C31	114.6 (7)	C50A—C51A—H51A	120.0
C33—C32—C31	121.4 (10)	C51A—C52A—C53A	120.0
C32—C33—C34	117.5 (14)	C51A—C52A—H52A	120.0
C32—C33—H33	121.2	C53A—C52A—H52A	120.0
C34—C33—H33	121.2	C54A—C53A—C52A	120.0
C35—C34—C33	119.0 (12)	C54A—C53A—H53A	120.0
C35—C34—H34	120.5	C52A—C53A—H53A	120.0
C33—C34—H34	120.5	C53A—C54A—N12A	120.0
C34—C35—C36	120.1 (11)	C53A—C54A—H54A	120.0
C34—C35—H35	120.0	N12A—C54A—H54A	120.0
C36—C35—H35	120.0	C54A—N12A—C50A	120.0
N8—C36—C35	121.8 (12)	C54A—N12A—Cu3	125.2 (8)
N8—C36—H36	119.1	C50A—N12A—Cu3	114.4 (8)
C35—C36—H36	119.1		
			
C7—N1—C1—C2	82.6 (8)	C43—N9—C37—C38	82.4 (10)
C13—N1—C1—C2	−152.2 (7)	C49—N9—C37—C38	−150.2 (8)
Cu1—N1—C1—C2	−35.7 (8)	Cu3—N9—C37—C38	−39.0 (9)
C6—N2—C2—C3	−2.4 (14)	N9—C37—C38—C39	−151.0 (7)
Cu1—N2—C2—C3	173.7 (7)	N9—C37—C38—N10	31.9 (10)
C6—N2—C2—C1	176.4 (8)	N10—C38—C39—C40	0.0
Cu1—N2—C2—C1	−7.5 (10)	C37—C38—C39—C40	−177.1 (10)
N1—C1—C2—N2	29.3 (10)	C38—C39—C40—C41	0.0
N1—C1—C2—C3	−151.9 (9)	C39—C40—C41—C42	0.0
N2—C2—C3—C4	1.3 (16)	C40—C41—C42—N10	0.0
C1—C2—C3—C4	−177.3 (10)	C41—C42—N10—C38	0.0
C2—C3—C4—C5	1.3 (19)	C41—C42—N10—Cu3	−173.6 (6)
C3—C4—C5—C6	−2.9 (19)	C39—C38—N10—C42	0.0
C2—N2—C6—C5	0.7 (14)	C37—C38—N10—C42	177.3 (9)
Cu1—N2—C6—C5	−174.8 (8)	C39—C38—N10—Cu3	174.5 (6)
C4—C5—C6—N2	1.9 (17)	C37—C38—N10—Cu3	−8.2 (8)
C13—N1—C7—C8	78.2 (8)	C37—N9—C43—C44	−157.2 (8)
C1—N1—C7—C8	−157.7 (7)	C49—N9—C43—C44	77.1 (10)
Cu1—N1—C7—C8	−39.2 (7)	Cu3—N9—C43—C44	−35.5 (9)
C12—N3—C8—C9	−2.6 (12)	N9—C43—C44—C45	−155.1 (7)
Cu1—N3—C8—C9	170.0 (7)	N9—C43—C44—N11	25.2 (11)
C12—N3—C8—C7	174.6 (8)	N11—C44—C45—C46	0.0
Cu1—N3—C8—C7	−12.8 (9)	C43—C44—C45—C46	−179.7 (10)
N1—C7—C8—N3	35.4 (10)	C44—C45—C46—C47	0.0
N1—C7—C8—C9	−147.4 (8)	C45—C46—C47—C48	0.0
N3—C8—C9—C10	1.7 (14)	C46—C47—C48—N11	0.0
C7—C8—C9—C10	−175.3 (9)	C47—C48—N11—C44	0.0
C8—C9—C10—C11	0.7 (15)	C47—C48—N11—Cu3	−179.6 (8)
C9—C10—C11—C12	−2.1 (16)	C45—C44—N11—C48	0.0
C8—N3—C12—C11	1.1 (13)	C43—C44—N11—C48	179.7 (9)
Cu1—N3—C12—C11	−170.4 (7)	C45—C44—N11—Cu3	179.7 (7)
C10—C11—C12—N3	1.3 (15)	C43—C44—N11—Cu3	−0.6 (8)
C7—N1—C13—C14	−158.3 (6)	C43—N9—C49—C50	−157.5 (9)
C1—N1—C13—C14	76.3 (8)	C37—N9—C49—C50	71.7 (10)
Cu1—N1—C13—C14	−40.7 (7)	Cu3—N9—C49—C50	−42.4 (9)
C18—N4—C14—C15	−1.3 (11)	N9—C49—C50—C51	−148.6 (7)
Cu1—N4—C14—C15	170.7 (6)	N9—C49—C50—N12	33.5 (11)
C18—N4—C14—C13	178.4 (7)	N12—C50—C51—C52	0.0
Cu1—N4—C14—C13	−9.6 (8)	C49—C50—C51—C52	−177.8 (10)
N1—C13—C14—N4	33.9 (9)	C50—C51—C52—C53	0.0
N1—C13—C14—C15	−146.4 (8)	C51—C52—C53—C54	0.0
N4—C14—C15—C16	−2.0 (13)	C52—C53—C54—N12	0.0
C13—C14—C15—C16	178.3 (8)	C53—C54—N12—C50	0.0
C14—C15—C16—C17	3.4 (13)	C53—C54—N12—Cu3	−174.5 (7)
C15—C16—C17—C18	−1.5 (13)	C51—C50—N12—C54	0.0
C14—N4—C18—C17	3.4 (11)	C49—C50—N12—C54	178.1 (9)
Cu1—N4—C18—C17	−167.5 (6)	C51—C50—N12—Cu3	175.4 (6)
C16—C17—C18—N4	−2.0 (12)	C49—C50—N12—Cu3	−6.5 (8)
C31—N5—C19—C20	−81.9 (9)	C49A—N9—C37A—C38A	−79.5 (16)
C25—N5—C19—C20	152.8 (7)	C43A—N9—C37A—C38A	157.0 (13)
Cu2—N5—C19—C20	35.6 (8)	Cu3—N9—C37A—C38A	43.1 (14)
C24—N6—C20—C21	1.3 (12)	N9—C37A—C38A—C39A	147.6 (10)
Cu2—N6—C20—C21	178.7 (6)	N9—C37A—C38A—N10A	−33.2 (16)
C24—N6—C20—C19	−176.9 (7)	N10A—C38A—C39A—C40A	0.0
Cu2—N6—C20—C19	0.5 (9)	C37A—C38A—C39A—C40A	179.2 (17)
N5—C19—C20—N6	−24.3 (10)	C38A—C39A—C40A—C41A	0.0
N5—C19—C20—C21	157.6 (7)	C39A—C40A—C41A—C42A	0.0
N6—C20—C21—C22	−2.3 (12)	C40A—C41A—C42A—N10A	0.0
C19—C20—C21—C22	175.8 (8)	C41A—C42A—N10A—C38A	0.0
C20—C21—C22—C23	1.7 (14)	C41A—C42A—N10A—Cu3	172.1 (10)
C21—C22—C23—C24	−0.2 (14)	C39A—C38A—N10A—C42A	0.0
C20—N6—C24—C23	0.3 (12)	C37A—C38A—N10A—C42A	−179.3 (15)
Cu2—N6—C24—C23	−176.6 (6)	C39A—C38A—N10A—Cu3	−172.6 (9)
C22—C23—C24—N6	−0.9 (14)	C37A—C38A—N10A—Cu3	8.2 (13)
C31—N5—C25—C26	154.8 (7)	C49A—N9—C43A—C44A	166.1 (15)
C19—N5—C25—C26	−80.3 (8)	C37A—N9—C43A—C44A	−78.4 (16)
Cu2—N5—C25—C26	37.9 (8)	Cu3—N9—C43A—C44A	31.6 (17)
C30—N7—C26—C27	−2.6 (12)	N9—C43A—C44A—C45A	157.4 (13)
Cu2—N7—C26—C27	176.1 (7)	N9—C43A—C44A—N11A	−28.8 (19)
C30—N7—C26—C25	−178.6 (7)	N11A—C44A—C45A—C46A	0.0
Cu2—N7—C26—C25	0.2 (9)	C43A—C44A—C45A—C46A	173 (2)
N5—C25—C26—N7	−26.0 (10)	C44A—C45A—C46A—C47A	0.0
N5—C25—C26—C27	158.0 (8)	C45A—C46A—C47A—C48A	0.0
N7—C26—C27—C28	2.9 (14)	C46A—C47A—C48A—N11A	0.0
C25—C26—C27—C28	178.6 (9)	C47A—C48A—N11A—C44A	0.0
C26—C27—C28—C29	−2.3 (15)	C47A—C48A—N11A—Cu3	172.5 (15)
C27—C28—C29—C30	1.6 (15)	C45A—C44A—N11A—C48A	0.0
C26—N7—C30—C29	1.8 (12)	C43A—C44A—N11A—C48A	−174.3 (19)
Cu2—N7—C30—C29	−176.7 (7)	C45A—C44A—N11A—Cu3	−173.1 (14)
C28—C29—C30—N7	−1.3 (14)	C43A—C44A—N11A—Cu3	12.6 (16)
C19—N5—C31—C32	159.0 (8)	C43A—N9—C49A—C50A	−92.7 (16)
C25—N5—C31—C32	−75.6 (9)	C37A—N9—C49A—C50A	155.9 (13)
Cu2—N5—C31—C32	40.8 (9)	Cu3—N9—C49A—C50A	39.6 (16)
C36—N8—C32—C33	−0.7 (14)	N9—C49A—C50A—C51A	151.3 (12)
Cu2—N8—C32—C33	−174.8 (10)	N9—C49A—C50A—N12A	−31.7 (18)
C36—N8—C32—C31	−176.3 (8)	N12A—C50A—C51A—C52A	0.0
Cu2—N8—C32—C31	9.6 (10)	C49A—C50A—C51A—C52A	176.9 (18)
N5—C31—C32—N8	−34.0 (11)	C50A—C51A—C52A—C53A	0.0
N5—C31—C32—C33	150.3 (11)	C51A—C52A—C53A—C54A	0.0
N8—C32—C33—C34	1 (2)	C52A—C53A—C54A—N12A	0.0
C31—C32—C33—C34	176.8 (13)	C53A—C54A—N12A—C50A	0.0
C32—C33—C34—C35	−1 (2)	C53A—C54A—N12A—Cu3	172.3 (13)
C33—C34—C35—C36	0 (2)	C51A—C50A—N12A—C54A	0.0
C32—N8—C36—C35	−0.6 (13)	C49A—C50A—N12A—C54A	−177.2 (16)
Cu2—N8—C36—C35	172.4 (8)	C51A—C50A—N12A—Cu3	−173.1 (12)
C34—C35—C36—N8	1.1 (18)	C49A—C50A—N12A—Cu3	9.7 (15)
